# Accelerated Enveloping Distribution Sampling to Probe the Presence of Water Molecules

**DOI:** 10.1021/acs.jctc.3c00109

**Published:** 2023-05-11

**Authors:** Oriol Gracia Carmona, Michael Gillhofer, Lisa Tomasiak, Anita De Ruiter, Chris Oostenbrink

**Affiliations:** † Institute for Molecular Modeling and Simulation, Department of Material Sciences and Process Engineering, University of Natural Resources and Life Sciences, Vienna, Muthgasse 18, 1190 Vienna, Austria; ‡ Christian Doppler Laboratory for Molecular Informatics in the Biosciences, University of Natural Resources and Life Sciences, Vienna, Muthgasse 18, 1190 Vienna, Austria

## Abstract

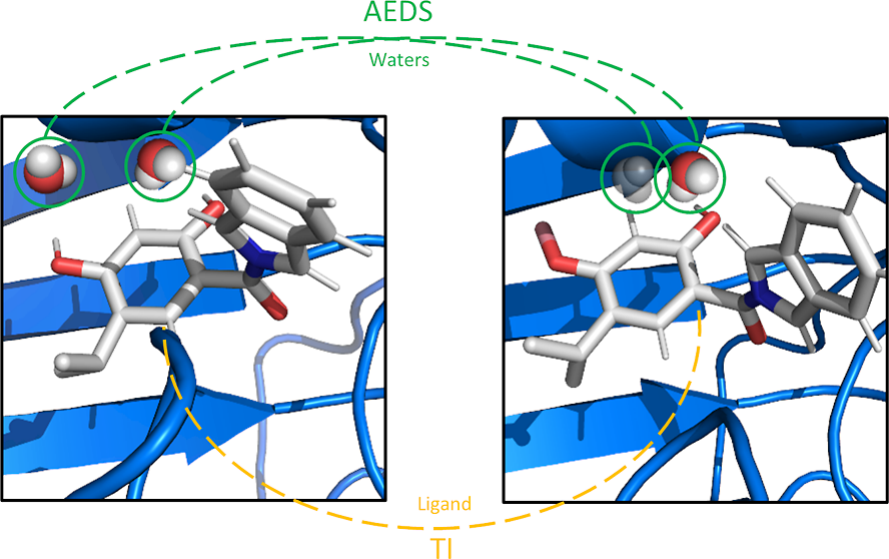

Determining the presence of water molecules at protein–ligand interfaces is still a challenging task in free-energy calculations. The inappropriate placement of water molecules results in the stabilization of wrong conformational orientations of the ligand. With classical alchemical perturbation methods, such as thermodynamic integration (TI), it is essential to know the amount of water molecules in the active site of the respective ligands. However, the resolution of the crystal structure and the correct assignment of the electron density do not always lead to a clear placement of water molecules. In this work, we apply the one-step perturbation method named accelerated enveloping distribution sampling (AEDS) to determine the presence of water molecules in the active site by probing them in a fast and straightforward way. Based on these results, we combined the AEDS method with standard TI to calculate accurate binding free energies in the presence of buried water molecules. The main idea is to perturb the water molecules with AEDS such that they are allowed to alternate between regular water molecules and non-interacting dummy particles while treating the ligand with TI over an alchemical pathway. We demonstrate the use of AEDS to probe the presence of water molecules for six different test systems. For one of these, previous calculations showed difficulties to reproduce the experimental binding free energies, and here, we use the combined TI–AEDS approach to tackle these issues.

## Introduction

In recent years, alchemical free-energy methods have increasingly found realistic application in drug design efforts.^1−3^ While, in the past, high computational costs to achieve sufficient sampling, inaccurate force fields, and other technical challenges led to major errors in free-energy calculations, by now, relative binding free energies can be calculated with an accuracy appropriate for drug discovery.^4,5^ Nevertheless, there are still challenges to be addressed.^6^ The occurrence of water molecules in the active site is one of them, and has sparked great interest due to its impact on the binding free energy.^7−12^ Hydrophobic effects have been estimated to be one of the major energetic contributions when it comes to ligand binding based on the fact that hydrophobic surface areas cover approximately 75% of the drug-binding pockets.^13^ Water molecules can modify the binding mode of different compounds by forming hydrogen bonds, which stabilize a specific conformational orientation of the ligand in the active site.^14^ There is a significant entropic effect, which should not be neglected since the movement of the water molecules in the buried site is much more restricted, whereas in solvents, they can move more freely.^14,15^ Therefore, it is necessary to treat the water molecules in the active site properly, especially if buried water molecules are present. In the case of infinite sampling, molecular dynamics (MD) should account for proper sampling throughout the simulation, including water molecule displacements and placements.^16^ However, in practice, MD simulations are restricted in sampling, usually ranging from ns to μs timescales. This time limitation becomes more pronounced when one wants to use MD in the lead optimization phase of the drug discovery process since multiple different compounds need to be evaluated.

To avoid having to simulate binding and unbinding events, which would require long simulation time scales, relative binding free energy (RBFE) techniques employing alchemical perturbations are commonly used instead.^17^ In RBFE calculations, the binding affinity between two similar ligands A and B is usually obtained by transforming ligand A into ligand B in both the bound state (ligand–protein complex in solution) and the free state (ligands in solution) over a set of non-physical alchemical intermediate states.^18^ However, when perturbing one ligand into another, the chemical properties in some parts of the ligands may change, which could lead to displacement, removal, or more room for water molecules. These factors are of great importance since, as already described, the restricted movement of water molecules can influence the overall accuracy in the relative binding free energies by strengthening different ligand–protein interactions.^5^ Additionally, due to the short simulation length used in alchemical methods, changes in water molecule preferences between both ligands to compare may lead to water molecules that are either trapped or unable to enter the active site. In the past, there have been various methods developed to accurately address these challenges.^9,19−27^ A few of these methods did already deliver good results and have been found to be used in RBFE workflows.^20^

Among these methods, the nonequilibrium candidate Monte Carlo (NCMC),^28^ when coupled to MD, as implemented in BLUES,^25^ can enhance the sampling in buried binding pockets, where water molecules can hop between energy basins. BLUES uses a defined spherical region around the ligand, where water hopping is allowed and additional relaxation steps are included to improve the acceptance of NCMC moves. Furthermore, there are grand canonical Monte Carlo (GCMC) methods which allow for the fluctuation by allowing insertion or deletion of water molecules in any given region. GCMC allows for the number of particles to fluctuate at a specific chemical potential and by using grand-canonical integration, one can incorporate the thermodynamics of the water molecules in calculating free energies of water networks.^23,24,26,29,30^ Recently, a combination of grand canonical nonequilibrium candidate Monte Carlo^22^ was developed, which exploits the smaller buffer region from GCMC and the relaxation step during insertions/deletions from NCMC. In general, these methods enhance the water molecule sampling process compared to normal MD, and GCMC seems to be more efficient in doing so.^31^

Additionally, when assuming that the correct amount of water molecules is known, a straightforward approach is to apply standard RBFE calculations such as thermodynamic integration (TI)^32^ or Bennett acceptance ratio^33^ while applying certain correction terms. For instance, if we consider that ligand A favors one water molecule in the active site and ligand B none, we can correct the RBFE difference by expanding the classical thermodynamic cycle to further include a correction term for removing one water molecule when perturbing ligand A into ligand B. This approach delivers only reasonable results when the correct number and the approximate position of the water molecules are known from experimental data (crystal structures) or other computational methods. It is important to note that water molecules reported in crystal structures are to be treated carefully since especially partially occupied hydration sites cloud the resulting electron density, which makes it hence more difficult to assign.^34^

In this work, we suggest to apply accelerated enveloping distribution sampling (AEDS) to probe solvation shells in the binding pocket by determining how favorable those water molecules are in the respective system. AEDS is a one-step perturbation method that uses a reference Hamiltonian, which is a combination of different Boltzmann-weighted end-state Hamiltonians.^35,36^ Using this method allows for sampling of the relevant end states. In this case, we define different amounts of water molecules being active. By using the acceleration parameters obtained from a solvent run, one can probe the presence of the molecule of interest in a quick and system-independent manner.^37^ Finally, we showcase the potential of using AEDS to treat water molecules in free-energy perturbation calculations by combining the AEDS method with standard TI and comparing them with the free energies obtained from other simulation setups.^38^

## Methods

### System Preparation

The benchmarks used for this work have been taken from the work of Wahl and Smieško.^7^ A total of six pairs of ligands were selected. Each pair of compounds features a minor chemical modification that leads to the displacement of one or two buried water molecules in the active site without producing any major conformational change. In this work, we designate these ligands using the same naming conventions as in the previous work.^7^ All the input protein systems were prepared using the available crystal structures in the protein databank. The used crystal structures are the following: Kung-13 pdb 3RLQ; Kung-16 pdb 3RLR;^39^ Davies-13b pdb 4FCP;^40^ Woodhead-1 pdb 2XAB; Woodhead-2 pdb: 2XJG;^41^ Cumming-4j pdb: 4DJW; Cumming-4b pdb: 4DJV;^42^ Chen-5d pdb: 3STD;^43^ Smith-8 pdb: 4ZLZ; and Smith-11 pdb: 4Z3V.^44^ All the water molecules present in the crystal structures were kept. For the systems for which no crystal structures were available, the starting coordinates were obtained by modifying the closest available crystals. In the case of Chen-3d, the structure of Chen-5d (3STD) was used to determine the protein and ligand orientation. The water molecule of the hydration shell was determined using the crystal structure of a closely related inhibitor, pdb: 5STD.^43^ This compound has a similar binding pose as the one under study and the same functional groups pointing to the hydration site, providing a reasonable guess for the starting coordinates of the water molecules. For system Davies-15b, the nitrile group was placed based on geometry and then allowed to relax during minimization. For all the pairs of the system that displayed no water molecules in their hydration sites, an additional dummy water molecule was placed by aligning the structure to the other member of the pair in which the water molecule was present. This dummy water molecule was used as the starting coordinates for the AEDS water molecule during the water probing runs. Both the protein and ligands were parametrized using the GROMOS 54a7 forcefield.^45^ Force-field parameters for the ligands were assigned based on analogy to similar functional groups in the force field and are available in the Supporting Information.

Short energy minimizations were performed for all the systems using the steepest-descent algorithm in a vacuum. The systems were placed in a periodic cubic water box of simple point charge (SPC) water^46^ molecules, leaving a minimum distance of 0.8 nm between the solute and the box walls. The water box positions were relaxed in a steepest descent minimization with position restraints on all the solute atoms and crystallographic water molecules. Counter ions were added by replacing water molecules with the most favorable electrostatic potential to reach charge neutralization using the program ion provided in the GROMOS++ package.^47^

Then, the systems were equilibrated with initial random velocities sampled from a Maxwell–Boltzmann distribution at 60 K and heated up to 300 K in five discrete steps. While heating up the system, position restraints on the solute atoms were reduced from 2.5 × 10^4^ to 0.0 kJ mol^–1^ nm^–2^. After temperature equilibration, an additional equilibration of 1 ns without position restraints and a constant pressure of 1 atm with an isothermal compressibility of 4.575 × 10^–4^ kJ^–1^ mol nm^3^ was performed. After equilibration, the atom positional root-mean-square deviation of the backbone atoms and ligand atoms was monitored to ensure that the systems were properly equilibrated.

All the production simulations were performed at a constant temperature of 300 K and pressure of 1 atm. Newton’s equations of motion were integrated using the leapfrog algorithm with a time step of 2 fs. The SHAKE algorithm^48^ was used to maintain the bond lengths at their optimal values. Long-range electrostatic interactions beyond a cut-off of 1.4 nm were truncated and approximated by a generalized reaction field^49^ with a relative dielectric permittivity of 61.^50^ Nonbonded interactions up to a distance of 0.8 nm were computed at every time step using a pairlist that was updated every 10 fs. Interactions up to 1.4 nm were computed at pairlist updates and kept constant in between.

### Water Probing

The accelerated version^35,36^ of the enveloping distribution sampling algorithm^51,52^ was used to probe water molecules by assessing the fractional occupancy of the water molecules of interest. The Hamiltonian of these water molecules was replaced by a reference state *H*_R_, which is obtained by combining the Hamiltonian of the different states of interest, *H*_*i*_. For this case, the Hamiltonian of an SPC water molecule (coupled state) and the Hamiltonian of a dummy water (dummy or decoupled state) are combined to form the reference Hamiltonian. For the cases in which two water molecules were probed at the same time, a reference state consisting of all the possible combinations of coupled and dummy water molecules was built instead, resulting in four end states (one with both water molecules coupled, two with one water molecule coupled, and one with two decoupled water molecules). To ensure equal sampling between all states, an energy offset, Δ*F*_*i*_^R^, is used to pull both energy minima at the same level (eq 1):^35^
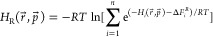
1with *RT* being the gas constant multiplied by the temperature (300 K). Building such a reference state results in an energy landscape with large energy barriers between the states that can hamper correct sampling of all the states of interest. To solve this issue, AEDS uses a harmonic boosting potential defined as follows (eq 2):^36^
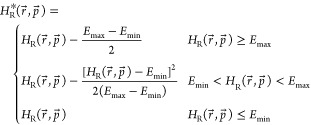
2where *E*_max_ is the energy maximum found when crossing between states and *E*_min_ is the low energy threshold used to define the acceleration range. The required parameters (*E*_max_, *E*_min_, and the offsets) can be obtained using a search algorithm.^36,37^ During the search algorithm, the end-states being sampled are monitored by comparing the values of . The state with the lowest value is considered to be currently sampled. The highest energy barrier observed when crossing through states is used as *E*_max_. *E*_min_ is calculated based on the fluctuations of the values of *H*_*i*_ in eq 1. The energy offsets are calculated as the free-energy difference between the accelerated end-state and the accelerated reference state.^36^ The end-state sampling times can be obtained by computing the weights of the different states over all of the simulation frames.^37^

To determine the acceleration parameters for the AEDS water molecules, a complete search of 100 ns was performed in bulk water. During the search runs, the offsets are estimated from the free-energy differences between the accelerated end-states and the reference state. The number of frames that will be used to determine the offsets are determined by a linearly increasing the memory decay function, going from 500 steps (1 ps) to 500,000 steps (1 ns). An additional production run of 100 ns was performed to corroborate that the selected acceleration parameters allowed quick fluctuations between states and roughly equal sampling times. The parameters obtained from this search in solvent were then used for all the hydration sites. To avoid the AEDS water molecule from leaving the active site due to its partially decoupled nature, distance restraints to the protein backbone were used, with a force constant of 1500 kJ mol^–1^ nm^–2^. The final acceleration parameters as well as the distance restraints used can be found in the Supporting Information.

### Extended TI

Relative binding free-energy calculations were performed for ligand pair Woodhead 1 and 2, bound to Heat shock protein 90 (Hsp90). The RBFE was calculated from thermodynamic cycles in which alchemical perturbations were performed twice: once for the ligand bound to the protein and once for the ligand in solvent. To infer the effect to the free energy of the buried water molecules, different combinations of water molecules being present or perturbed were used alongside the ligand perturbation, resulting in the following setups: both crystallographic water molecules (2144, 2295) being present unperturbed, both crystallographic water molecules being absent, both crystallographic water molecules being perturbed (going from the coupled to the decoupled state), and both water molecules being present and water 2144 being perturbed. For all the perturbations in which a water molecule was perturbed in addition to the ligand, perturbed distance restraints coupled to λ were used, ranging from a force constant of 0 kJ mol^–1^ nm^–2^ at the λ window with the coupled water molecule to 1500 kJ mol^–1^ nm^–2^ at the λ window in which the water molecule was decoupled. Each perturbed water molecule was restrained by a harmonic distance restraint with a reference distance of 0 relative to a single virtual site, which was defined using the center of geometry of the selected protein atoms to avoid indirectly bonding those atoms. The free energy of removing an SPC water molecule from bulk water (26.8 kJ/mol) was added to the resulting ΔΔ*G* from the alchemical perturbations for each water molecule that was perturbed alongside the ligand. Additionally, the effect of the distance restraint was corrected analytically using eq 3:^53,54^
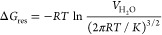
3where *K* is the force constant used for the distance restraining and  is the volume of one SPC water molecule in bulk water at the SPC density (3.0777 × 10^–2^ nm^3^).

All the perturbations were performed using the extended TI methodology^55^ and a single topology approach.^56^ In extended TI, the derivatives of the Hamiltonian with respect to λ are predicted for a number of additional λ-values than actually simulated, leading to smoother curves and more precise free-energy estimates.^55^ The number of precalculated λ-points was set to 101. A soft-core potential was used for the Lennard-Jones (LJ) and Coulomb + reaction field (CRF) interactions, with soft-core parameters α_LJ_ = 0.5 and α_CRF_ = 0.5 nm^2^ for the atoms being perturbed. A total of 11 equally spaced λ-values of 20 ps of equilibration and 1 ns of production were used. Both the electrostatic and Lennard-Jones interactions were perturbed at the same time. Backward simulations for all the perturbations were performed starting from the last frame of λ-point 1.0.

### Thermodynamic Integration-Accelerated Enveloping Distribution Sampling

The AEDS algorithm was extended to make it compatible with the existing TI code. In the TI-AEDS simulations, the water molecule of interest was treated with the AEDS algorithm while the ligand perturbation was treated with TI. The free energy difference can be calculated according to eq 4:
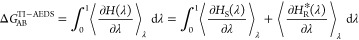
4in which *H*_S_ corresponds to the Hamiltonian of the system excluding the AEDS reference state and *H*_R_^*^ correspond to the accelerated AEDS Hamiltonian. The partial derivative of the accelerated AEDS Hamiltonian with respect to λ can be obtained from eq 5:
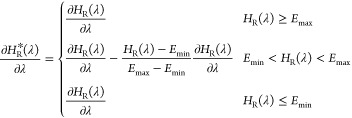
5The derivatives of the AEDS Hamiltonian, *H*_R_, are obtained from eq 6
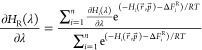
6

The performed TI-AEDS simulations follow a similar simulation setup to the one described for the extended TI simulations. The number of λ windows was increased to 41 equally spaced λ-points of 5 ns each in order to achieve converged free energies. The increase in λ-points was needed since the current TI-AEDS implementation is not compatible with the existing extended TI one which provides convergence with less simulated λ-points. The perturbation topology was changed to a double topology approach to avoid perturbing bonds, and the distance restraint was applied constantly on the AEDS water molecule instead of using a distance restraint coupled to λ. Additional TI simulations following the same setup as described but treating the water molecule with TI were performed. These simulations were used to be able to further assess the accuracy and consistency of the TI-AEDS simulations by comparing not only the final ΔΔ*G* of binding but also the Δ*G* of each individual path.

## Results

### AEDS Water Probing

To test the capabilities of AEDS to probe the water molecules of interest by assessing their fractional occupancy, a previously reported set of benchmark systems designed to study changes in solvation of active sites was used.^7^ This benchmark consists of six pairs of compounds on four different proteins, in which each pair of compounds contains a small modification which results in a different number of buried water molecules present in the active site.^7,8,39−44^ Incorrect sampling of the solvation shell of these ligands leads to wrong free energy of binding estimations and to significant hysteresis between replicates depending on the initial solvation.^7,8^

The initial structures were prepared as described by Wahl and Smieško^7^ but with the water molecules known to be different between both members of a pair of compounds replaced by AEDS waters. An AEDS water is an artificial molecule consisting of the combination of the Hamiltonians of a water molecule and a dummy water molecule. Thanks to this, the AEDS water has the capabilities of switching between the coupled and decoupled states (dummy).

However, the resulting energy barriers from combining both Hamiltonians cannot be overcome during a conventional simulation, which would lead to simulations stuck in one state, and even if this barrier is crossed, the different depth of the energy minima of both states would lead to simulations that spend most of the time in one end-state only. To overcome this limitation, AEDS uses an energy offset to pull the minima to a similar height, ensuring a more equal sampling of the states of interest, and then defines an acceleration region in which a boosting potential is added to decrease the energy barriers,^35,36^ achieving, in this way, fast switching between the end-states of interest. In the standard AEDS approach, since these offsets and acceleration range are system-dependent, they can be obtained during a preliminary search run.^36^ First, a complete search of all the AEDS parameters is performed in bulk water. Then, a search of only the energy offsets is performed in the protein, but keeping the acceleration range to the values obtained from the search in bulk water to allow for a faster convergence.^36,37^ In this work, the AEDS methodology is used as a screening tool instead. For this approach, the acceleration parameters are searched only once in bulk water and then used in all the systems. Using the parameters optimized in bulk water on the protein results in unequal sampling of the different end-states, in which the more energetically favorable end-states are sampled more often. This shift in observed sampling, when compared to the sampling in bulk water, can be used to infer the likelihood of the presence of the molecules of interest.^37^ In this work, we use this screening capability of AEDS to probe or asses the fractional occupancy of buried water molecules by substituting them with AEDS waters. In summary, the AEDS water is able to freely shift between coupled and decoupled states during the simulation based on its surroundings, behaving similar to a chemostat.^37^

For the case of the AEDS waters, since the molecule to be probed is always the same, a water molecule, the acceleration parameters obtained in bulk can be used in a system-independent manner to probe hydration sites of any protein. AEDS search runs of 100 ns were performed to guarantee that the obtained acceleration parameters are well converged. The obtained acceleration parameters were then validated with 100 ns AEDS production runs, see the Supporting Information. The obtained acceleration parameters allowed for equal sampling of all the end-states of interest with rapid fluctuations between them with and an average lifetime of 6.7 ± 1.3 ps for both states in the simulation with one AEDS water and 5.5 ± 3.5 ps for the simulation with two AEDS water molecules. The obtained free energy of solvation using these parameters was 26.6 ± 0.5 kJ/mol, in good agreement with the free energy of solvation obtained with extended TI (26.8 ± 0.3 kJ/mol). Each ligand–protein complex under study was then simulated individually with AEDS water molecules for 10 ns. The sampling times of the coupled and decoupled states for each AEDS water molecule was monitored by using their respective contribution to the energies of every configuration in the simulation.^37^

**Figure 1:**
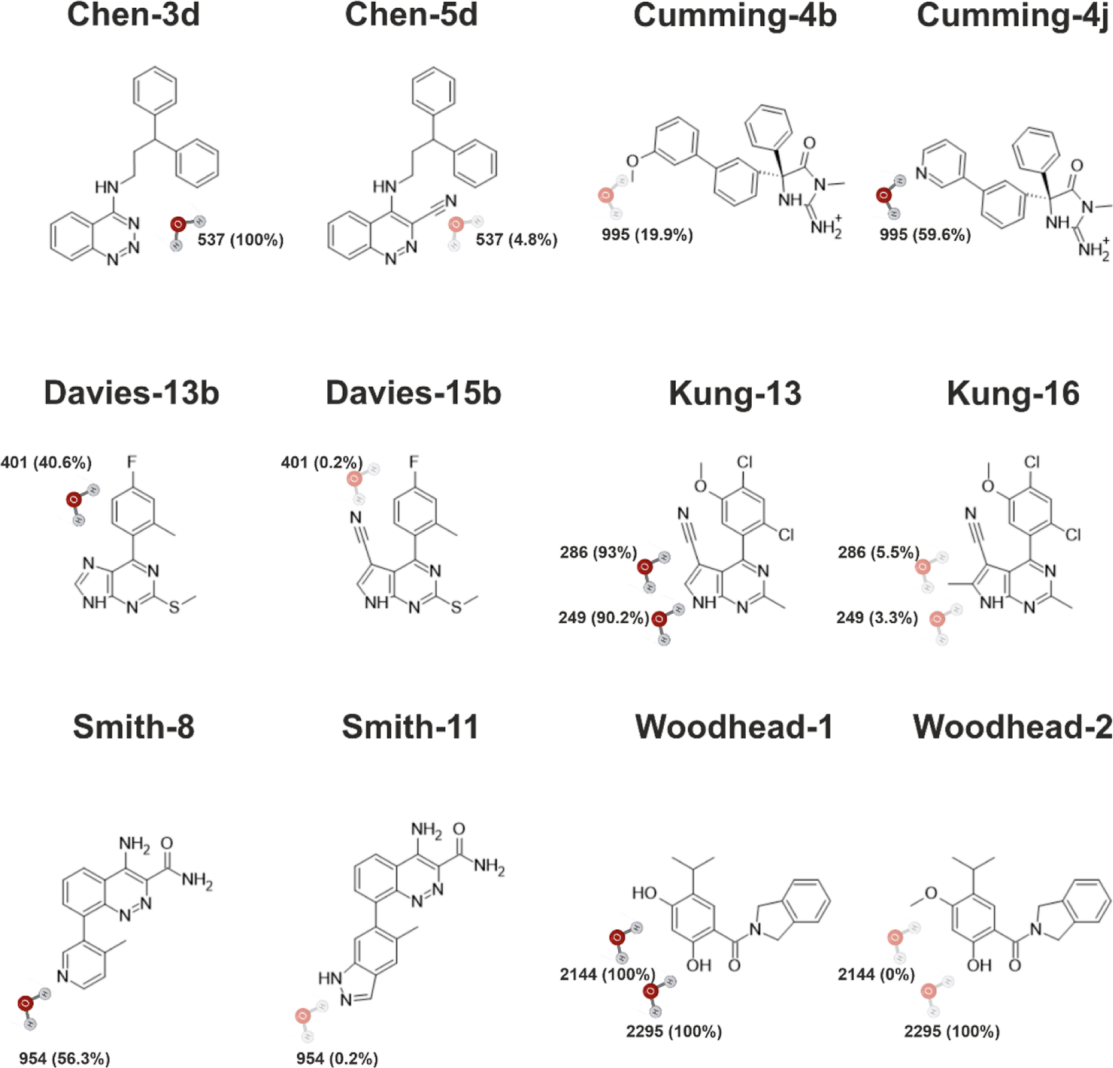
Schematic diagram of the pairs of compounds under study with the crystallographic water molecules that are found to be displaced in one of the pairs.^7^ The number of the water molecule indicates its pdb residue number, while the percentage in brackets indicates the percentage of simulation time in which the active state was sampled, determined by assessing the weight that the coupled water molecule had in the energy of every configuration of the simulation.^37^ The water molecules that are shown with intense colors indicate that the water molecules were observed in the crystal structure for that ligand, while the water molecules depicted with faded colors indicate those water molecules that are not present in the available crystal structures. For the compound pair Chen-3d–Chen-5d, the addition of the nitrile group produces a displacement of water 537 observed in the crystal structure of a closely related inhibitor (5STD).^43^ For the pair Cumming-4b–Cumming-4j, the change of the nitrogen of the pyridine ring of Cumming-4j to a methoxy group produces the displacement of water 995, pdbs: 4DJW, 4DJV.^42^ For the pair Davies-13b–Davies-15b, the addition of the nitrile group produces a displacement of the water 401 observed in the crystal structure of Davies-13b (4FCP). For the pair Kung-13–Kung-16, the addition of a methyl group produces the displacement of two water molecules (249, 286) from the active site, pdbs: 3RLQ, 3RLR.^39^ For the pair Smith-8–Smith-11, the extension to an indazole group produces the displacement of water 954, pdbs: 4ZLZ, 4Z3V.^44^ Finally, for the pair Woodhead-1–Woodhead-2, the conversion from a phenol moiety to a methoxyphenyl produces the displacement of two crystallographic waters (2144, 2295), pdbs: 2XAB, 2XJG.^41^ However, for this system, the AEDS simulations suggest that only water 2144 is displaced instead of both.

The resulting sampling times closely matched the observed number of water molecules in the crystal structures of the different pairs of ligands under study,^39−44^ as depicted in Figure 1. Overall, the simulations were already converged before reaching the 10 ns mark, allowing to infer the likelihood of the water molecule to be in the active site with as little as 5 ns, showcasing the speed in which this method can probe hydration sites. The convergence plots of the water molecule sampling times for each system can be found in the Supporting Information.

The pair of compounds (Chen-3d–Chen-5d),^43^ in which there is one water molecule being displaced from the active site, shows a clear preference to the number of water molecules seen in the crystal structure, with the water molecule being present 100% of the simulation time for compound Chen-3d and only 4.8% of the simulation time for compound Chen-5d.

**Figure 2:**
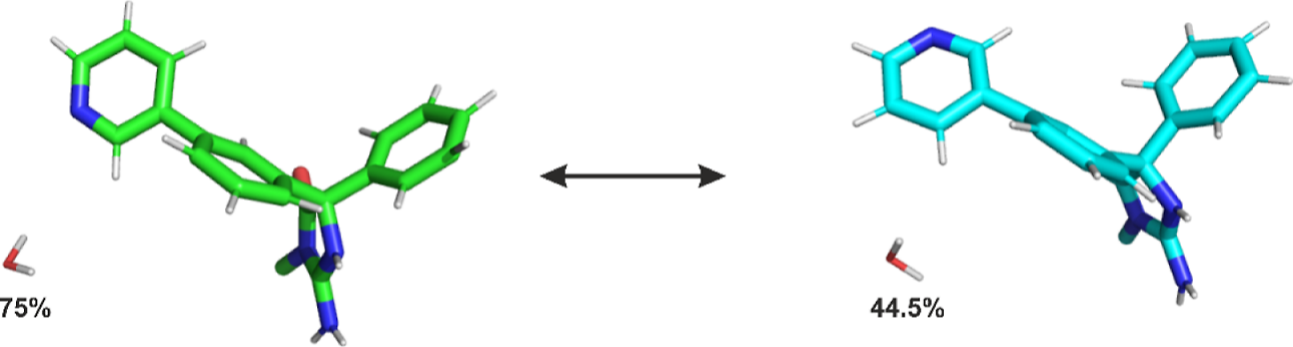
Stick representation of the conformations observed for ligand Cumming-4j over 10 ns of the AEDS probing run, with the orientation matching the one observed in the crystal structure in green and the conformation with the pyridine ring flipped in cyan. The percentage indicates the sampling times of the activated state of the water molecule being probed over all the frames in which the ligand showed that given conformation.

The pair of compounds (Cumming-4j–Cumming-4b)^42^ showed a more diverse presence of water molecules. Cumming-4j showed 59.6% of sampling time with the water molecule being present, and this water molecule showed clear preference for Cumming-4j when compared to Cumming-4b, in which the water molecule was observed only 19.9% of the simulation time. A similar preference can be observed from the free energies, with a binding free energy of the water molecule of 0 kJ/mol for Cumming-4j and an unfavorable binding free energy of 4 kJ/mol for the water molecule in the presence of Cumming-4b. This pair of compounds is known to be challenging to evaluate since the pyridine ring of ligand Cumming-4j, with which the water molecule being probed interacts, can flip during the simulation.^7^ To better account for the conformational change of Cumming-4j, the presence of the AEDS water was traced as a function of the ring orientation observed, Figure 2. When the orientation of the ring matched the one observed in the crystal structure, the AEDS water molecule showed a clear preference for the active state, with the water molecule being present 75% of the time in comparison to 44.5% of the time when the pyridine ring was rotated, showing that the AEDS water can successfully adapt to a shifting environment. As the flip of the pyridine ring was observed only once, the simulation does not allow for quantitative statements about the relative free energies of the two conformations. To obtain a full estimate of the fractional occupancy of the water molecule, the energetic and entropic contributions of the ligand conformational change would need to be included.

The pairs (Davies-13b–Davies-15b)^40^ and (Smith-8–Smith-11)^44^ also showed a partial hydration for the ligands in which a water molecule is expected to be present, Davies-13b and Smith-8, with the coupled water state being present 40.6 and 56.3% of the simulation times, respectively. The obtained binding free energies for the water molecule in Davies-13b and Smith-8 systems were slightly unfavorable, with 6 and 3 kJ/mol, respectively. Although longer simulations would be needed to obtain converged free energies, the obtained values also hint that the water molecule probed has a similar preference to be in the solvent as in the active site. When compared to their counterparts, there is a clear difference in the water molecule preference, with compounds Davies-15b and Smith-11 only having the water molecule present for less than 1% of the total simulation time. In the case of Davies-13b, it is difficult to assess the real occupancy of that hydration site since there is no crystal structure resolved for this ligand. However, for Smith-8, there is a crystal structure available with a clear occupancy for that water molecule.^44^ Occurrence of the water molecules around 50% indicate that the water molecules behave comparably to bulk water and would have a binding affinity of roughly 0 kJ/mol. A consequence could be that the impact of the water molecule for the binding of those ligands is moderate.^7^

The pair of compounds (Kung-13–Kung-16), in which two water molecules are displaced, showed a clear preference for the coupled state of the AEDS water that matched the one observed on the crystal structures and electron density.^39^ For compound Kung-13, both water molecules were present during most of the simulation time (83.5%), with water molecule 289 present for 93% and water molecule 249 for 90.2% of the simulation time, while compound Kung-16 showed mostly no water molecules present with 91.2% of the time having no water molecules in those hydration sites.

Finally, the pair Woodhead-1–Woodhead-2 was the only pair of compounds for which the AEDS water preferences did not match the experimentally observed ones. For Woodhead-1, both water molecules were present for most of the simulation time as seen in the crystal. However, for Woodhead-2, one of the water molecules, water molecule 2295, was present for closely 100% of the simulation time; when in the reported crystal structure, Woodhead-2 has no water molecules in that hydration site.

### TI Simulations

To study the discrepancy in the water molecule preferences of the Woodhead-1–Woodhead-2 pair of compounds in more detail, a set of four TI simulations using the extended TI algorithm were performed.^55^ For all the sets of simulations, both forward and backward simulations were performed to identify possible sources of hysteresis due to the simulation setup. Each set of TI calculations used a different combination of the relevant water molecules in the active site being present or perturbed in addition to the perturbation of the ligand. One calculation was performed with the two water molecules being present continuously, without perturbing them, one calculation while perturbing both relevant water molecules at the same time (reproducing what is observed in the crystal structures), one calculation with no water molecules present on either end-state, and one calculation perturbing only the water molecule 2144 (the water molecule that AEDS predicted to be present).

**Figure 3:**
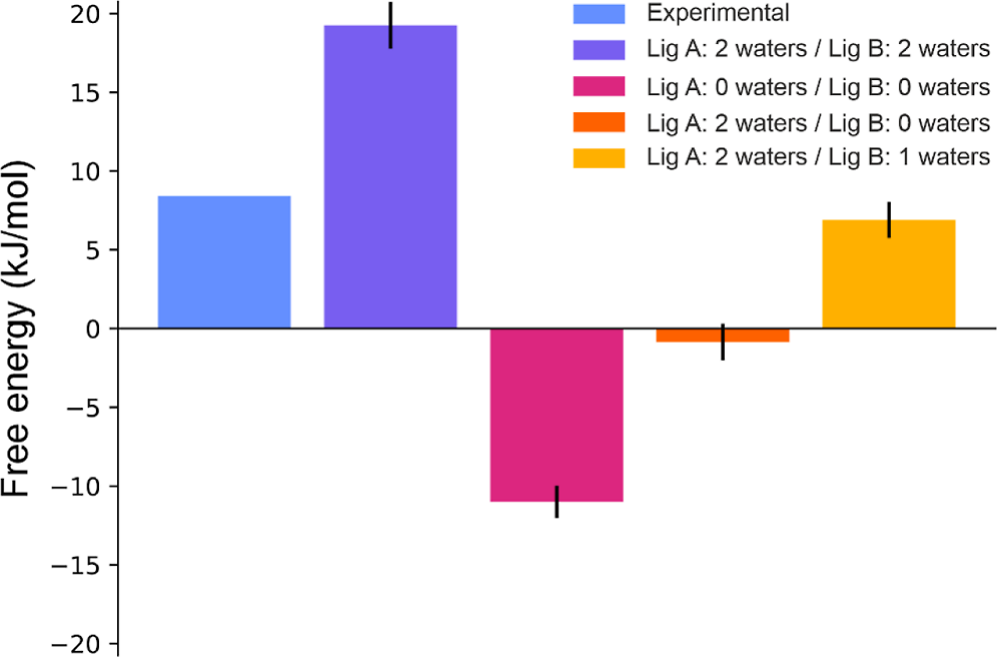
ΔΔ*G* of binding for Woodhead-1 (lig A) → Woodhead-2 (lig B). Experimental value of 8.4 kJ/mol (experimental); calculated ΔΔ*G* of binding for which the water molecules in both hydration sites were kept, 19.2 ± 1.5 kJ/mol (Lig A: 2 waters/Lig B: 2 waters); ΔΔ*G* of binding without water molecules present in the hydration sites, −11 ± 1 kJ/mol (Lig A: 0 waters/Lig B: 0 waters); ΔΔ*G* of binding in which both water molecules of the relevant hydration sites were perturbed alongside the ligand, reproducing what is observed in the crystal structures, −1.2 ± 1.2 kJ/mol (Lig A: 2 waters/Lig B: 0 waters); ΔΔ*G* of binding in which only the water molecule predicted to be relevant by the AEDS probing runs (water 2144) was perturbed alongside the ligand, 6.9 ± 1.1 kJ/mol (Lig A: 2 waters/Lig B: 1 water).

The TI setup that produced the closest values to the experimental relative free energy of binding was when water molecule 2144 was perturbed alongside the ligand, with a calculated ΔΔ*G* of 6.9 ± 1.1 kJ/mol, only 1.5 kJ/mol away from the experimental one of 8.4 kJ/mol.^41^ All the other sets of simulations produced free energies of more than 5 kJ/mol away from the experimental ones, see Figure 3. All reported values correspond to the average over the forward and backward simulations, including all the relevant corrections [inserting water molecules that are removed from the active site into bulk water and removal of the distance restraint on a decoupled water molecule (eq 3)] as described in the methods section. In the simulation in which no water molecules were present, only the forward simulation was used due to the high hysteresis observed, 5 kJ/mol. All the other simulations showed hysteresis of less than 2 kJ/mol. The high hysteresis for this particular set of simulations is also an indicator that the presence of a water molecule at those hydration sites is of relevance.

This suggests that the ligand Woodhead-2 would have an extra water molecule in its solvation shell that forms hydrogen bonds with both residue ASP 93 and the ligand. This is in contrast to the crystal structure, where there are no water molecules present at that site.^41^ One explanation for why this water molecule is not observed in the crystal structure of Woodhead-2 could be that this water molecule in particular is quite flexible, which would lead to a lack of electron density in the crystal structure. Alternatively, the current computational results could be an artifact of the forcefield used.^57,58^ Regardless of the underlying reason, the number of water molecules that leads to TI results closer to the experimental RBFE matches the water preferences obtained from the AEDS run, showing that the AEDS water sampling is consistent with the forcefield used.

### Thermodynamic Integration–Accelerated Enveloping Distribution Sampling

In addition to probing the presence of water molecules in different environments, AEDS waters can also be used in combination with other free-energy techniques to allow for a dynamic sampling of the water molecules of interest over time. To do so, the existing AEDS algorithm was expanded to interact with the TI algorithm of GROMOS. The ligand Woodhead-1 was perturbed to Woodhead-2 using the TI algorithm, while water molecule 2114, the one found to behave differently between both ligands, was replaced with an AEDS water and allowed to fluctuate.

**Figure 4:**
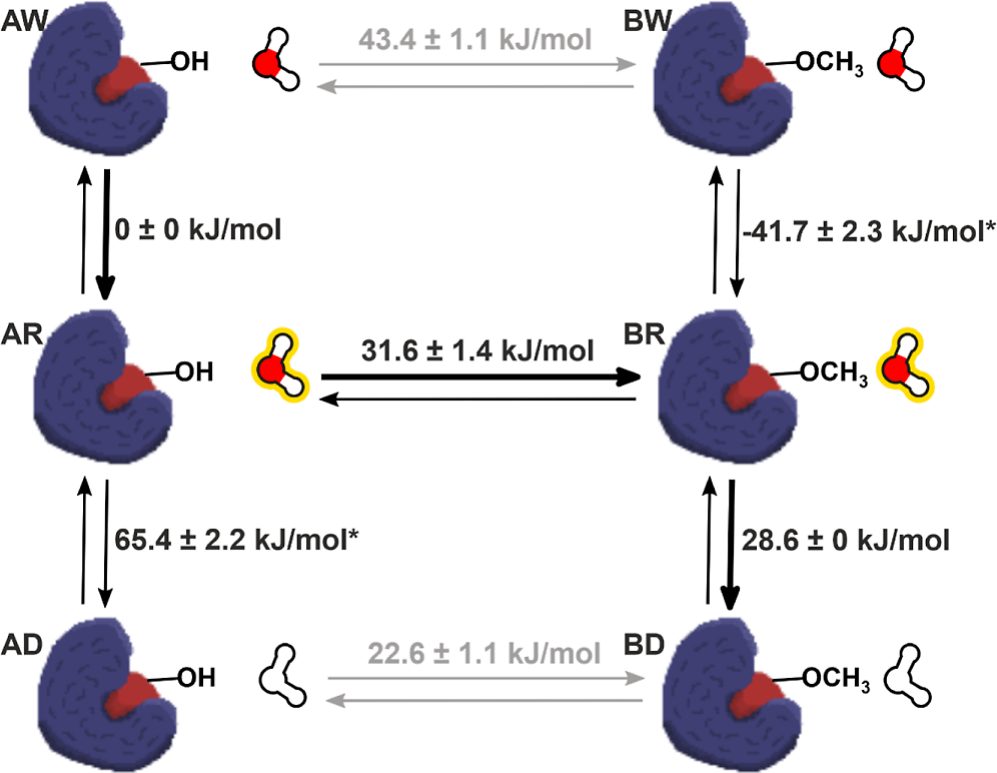
Schematic representation of the thermodynamic cycle obtained by using the described TI–AEDS setup, with the obtained Δ*G* along every path. The protein bound to Woodhead-1 on the left and to Woodhead-2 on the right. The water molecule with a yellow overlay represents the AEDS water reference state, while the blank water molecules represent the dummy state. The paths and values obtained directly from the performed simulations are depicted in black, and the values predicted through reweighting are shown in gray. The path that corresponds to Δ*G* of perturbing Woodhead-1 to Woodhead-2 bound in protein is highlighted by the arrows in bold; the obtained Δ*G* closely matches the TI-only simulation of the same path, 60.2 ± 1.4 and 59.9 ± 1.5 kJ/mol, respectively. The values marked with an asterisk correspond to unreliable free energies due to lack of sampling of that water state for that given λ window.

By combining both AEDS and TI, one can obtain the thermodynamic cycle shown in Figure 4, where the simulated perturbation, Woodhead-1 to Woodhead-2 with the presence of an AEDS water, corresponds to the middle line of the diagram. To then obtain the RBFE of interest, it is necessary to compute the free energies of going from the AEDS water to the dummy state and to the coupled water state for both ligands of interest. These free energies can be obtained from λ windows 0 and 1, respectively, using a one-step perturbation approach on the AEDS energies. The final free energy of perturbing Woodhead-1 to Woodhead-2 corresponds to the main AEDS water states sampled at λ window 0.0 and 1.0. In the case of this test system, it is the path going from the water molecule 2144 being present at λ 0.0 (Woodhead-1) to the dummy water molecule at λ 1.0 (Woodhead-2).

To better compare the free energy obtained with the TI–AEDS setup, an additional TI simulation perturbing both water molecule 2144 and the ligand was performed, this time without the extended TI algorithm and using the same setup as the TI–AEDS run. The free energies obtained from the upper left-hand corner to the lower right-hand corner of the cycle in Figure 4 closely match each other, 60.2 ± 1.4 kJ/mol for the TI–AEDS simulation and 59.9 ± 1.5 kJ/mol for the TI-only simulation. In addition, both free energies correctly match the final relative free energy of binding obtained with extended TI once the perturbation of the ligand in the solvent and corrections are factored in, 7.1 ± 1.4 kJ/mol for the TI–AEDS run and 6.9 ± 0.9 kJ/mol for the extended TI one.

**Figure 5:**
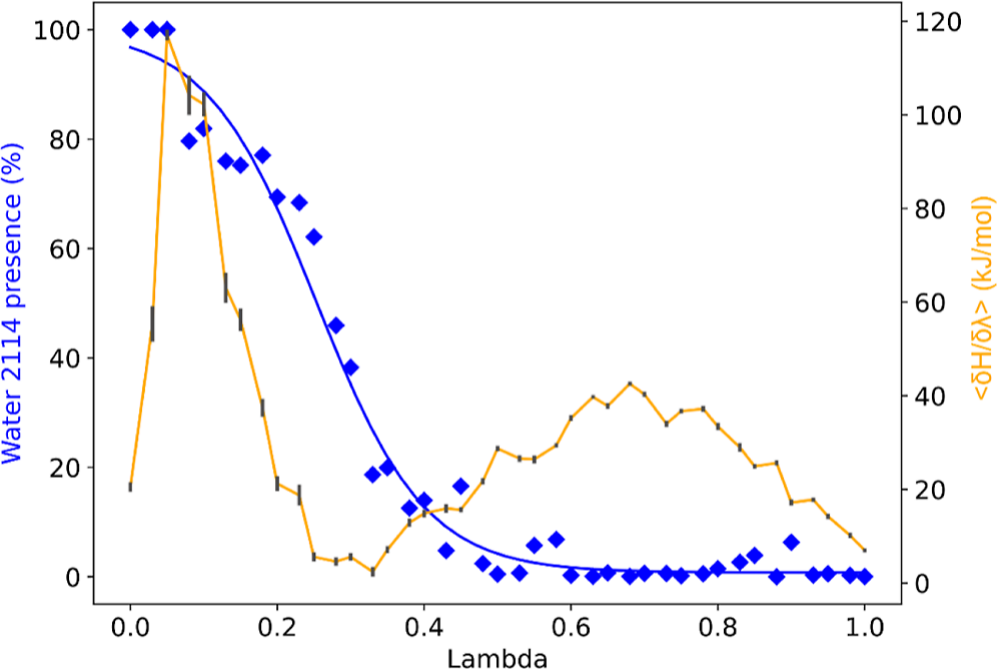
⟨∂*H*/∂λ⟩ profile of the perturbation from Woodhead 1 to Woodhead 2 treating water 2144 with AEDS in orange with the standard error in black. Percentage of time spent during the simulation with the AEDS water 2144 being active for each of the simulated λ windows, blue diamonds; the blue line is a curve fitted over the data points to aid the readability.

Additionally, the presence of water can also be traced along the different λ simulations, allowing for a comparison of the ⟨∂*H*/∂λ⟩ profile with the presence of the water molecule (Figure 5). At the point in which the ⟨∂*H*/∂λ⟩ reaches the first peak (at λ = 0.05), the water presence starts to drop from nearly 100% of the time and decreases over the λ windows until finally reaching ∼0% at the second peak (at λ = 0.75) of ⟨∂*H*/∂λ⟩. This strongly suggests that the AEDS water can successfully respond to small changes in the environment.

From the performed TI–AEDS simulation, it is also possible to predict the free-energy difference of paths that are not directly simulated, such as perturbing Woodhead-1 to Woodhead-2 with the water molecule 2144 always present or absent, by reweighting the simulations using the corresponding time series, eq 7
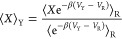
7where *X* is the property to reweight (∂*H/*∂λ), *V*_y_ is the energy time series of either the water molecule being coupled or in the dummy state, and *V*_R_ is the energy time series of the AEDS reference state.

To better assess the reliability of the reweighted simulations, two additional TI runs, one in which the water molecule 2144 was always present and one in which it was in the dummy state, were performed using the same setup as in the TI–AEDS simulations. Both TI simulations were in good agreement with the results obtained from the reweighted TI–AEDS simulations, with differences below the 2.0 kJ/mol range (43.4 ± 1.1 kJ/mol for TI–AEDS and 43.2 ± 1.6 kJ/mol for TI only, for the perturbation with water 2144 always present, 22.6 ± 1.1 kJ/mol for TI–AEDS and 20.8 ± 1.4 kJ/mol for TI only, for the perturbation in which water molecule 2144 was always in the dummy state).

**Figure 6:**
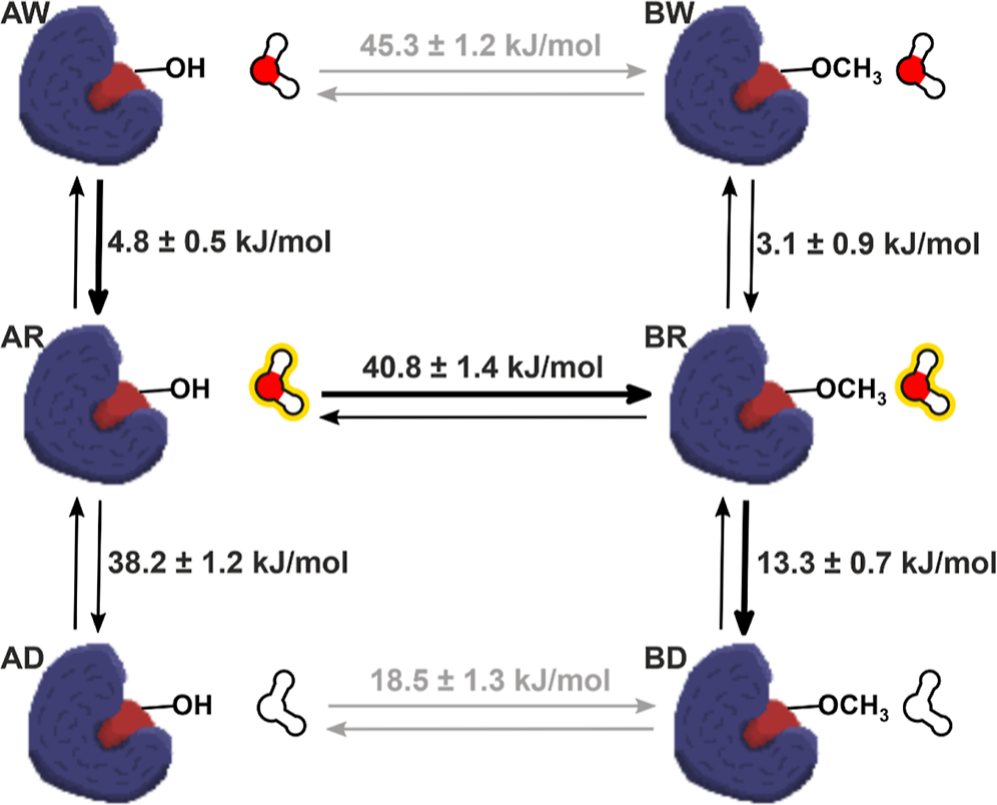
Schematic representation of the thermodynamic cycle obtained by using the described TI–AEDS setup with optimized offsets, with the obtained Δ*G* along every path. Protein bound to Woodhead-1 (A) on the left and to Woodhead-2 (B) on the right. The water molecule with a yellow overlay represents the AEDS water reference state (R) while the blank water molecules represent the dummy state (D). The paths and values obtained directly from the performed simulations are depicted in black, and the values predicted through reweighting are shown in gray. The path that corresponds to Δ*G* of perturbing Woodhead-1 to Woodhead-2 bound in protein is highlighted by the arrows in bold.

Finally, one can compute the cycle closures for the whole obtained cycle. The cycle closures obtained are poor, having as high as 57.7 kJ/mol of error due to incorrect estimation of the free energy of solvation of water molecule 2144 in the active site at λ 0.0 and 1.0. This high error stems from the sampling observed of the AEDS water in those λ windows. Since the other water state is never seen, the estimation of the free energy becomes unreliable, as indicated with an asterisk in Figure 4. One solution would be to set up a free-energy perturbation to estimate the energy of solvation at those λ values. Another option would be to optimize the acceleration parameters such that one achieves sampling of both AEDS water end-states. In this way, one should be able to get accurate free-energy differences of the whole cycle. To do so, two additional AEDS search runs were performed for the AEDS water 2144 with Woodhead-1 as the ligand and another with Woodhead-2. Because the offsets required to enforce equal sampling of both water states in both cases are different, 35 and −25 kJ/mol, respectively, the offset needs to be changed through the different λ windows. In this work, we used a linear interpolation in function of λ, resulting in the following reference Hamiltonian, eq 8
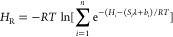
8where *S* is the slope used in the linear interpolation of the offset (*S*_0_ = 0 kJ/mol; *S*_1_ = 60 kJ/mol) and *b* is the constant term of the linear equation (*b*_0_ = 0 kJ/mol, *b*_1_ = 35 kJ/mol). In addition to the changing offset, the acceleration range was increased, and additional distance restraints to water molecule 2295 were used to avoid the binding site from distorting due to the forced presence or absence of the water molecule and the increased acceleration. In the evaluation of ∂*H*/∂λ, the additional λ-derivative term, due to eq 6 was included. The relative binding free energies obtained from this new setup were in good agreement with the previous TI–AEDS and TI simulations while also providing reasonable cycle closures of the whole cycle, below 3.0 kJ/mol, Figure 6. Table 1 compares the different free-energy terms of Figures 4 and 6 to the values obtained when using TI only.

**Table 1:** Δ*G* Values Obtained in kJ/mol by the TI–AEDS Simulation with Universal Water Parameters (TI–AEDS); TI–AEDS Simulation with Optimized Offsets (TI–AEDS Optimized); and TI-Only Simulations in Which all the Perturbations Were Performed Using the Standard TI Methodology (TI)^a^

perturbation	TI–AEDS (kJ/mol)	TI–AEDS optimized (kJ/mol)	TI (kJ/mol)
Δ*G*_AW → BW_	43.4 ± 1.1^b^	45.3 ± 1.6^b^	43.2 ± 1.2
Δ*G*_AR → BR_	31.6 ± 1.4^c^	40.8 ± 1.4^c^	
Δ*G*_AD → BD_	22.6 ± 1.1^b^	18.5 ± 1.4^b^	20.8 ± 1.3
Δ*G*_AW → BD_	60.2 ± **1.4**	58.9 ± **1.5**	59.9 ± **1.6**
Δ*G*_AW → AR_	0 ± 0^c^	4.8 ± 0.5^c^	
Δ*G*_AR → AD_	65.4 ± 2.2^c^	38.2 ± 1.2^c^	
Δ*G*_BW → BR_	–41.7 ± 2.3^c^	3.1 ± 0.9^c^	
Δ*G*_BR → BD_	28.6 ± 0^c^	13.3 ± 0.7^c^	

a For simplicity, Woodhead-1 is referred to in the subindex as state A and Woodhead-2 as state B. The water states are D for the dummy water molecule, R for the AEDS water, and W for the coupled water molecule. The final Δ*G* of perturbing Woodhead-1 to Woodhead-2 bound to protein corresponds to the values highlighted in bold (Δ*G*_AW → BD_). Cycle closure for three cycles is confirmed for the TI–AEDS simulation with optimized offsets. Cycle closures: Δ*G*_AW → BW_ + Δ*G*_BW → BR_ – Δ*G*_AR → BR_ – Δ*G*_AW → AR_ = **2.8** kJ/mol, Δ*G*_AW → BW_ + Δ*G*_BW → BR_ + Δ*G*_BR → BD_ – Δ*G*_AD → BD_ – Δ*G*_AR → AD_ – Δ*G*_AW → AR_ = **0.2** kJ/mol, Δ*G*_AR → BR_ + Δ*G*_BR → BD_ – Δ*G*_AD → BD_ – Δ*G*_AR → AD_ = **2.6** kJ/mol.

b Predicted values trough reweighting.

c Values are not directly comparable since the reference AEDS state used is different.

However, since usually one is not interested in describing the whole cycle but rather in obtaining the free energy of the process of interest, this parameter optimization is not necessary and one can use the parameters obtained in water in a transferable manner to any protein. Overall, the tested setups provided free energies with good agreement between each other.

## Conclusions

The change in water networks on the active site can have a significant impact on the calculation of relative protein–ligand binding free energies. This issue becomes more prevalent in alchemical perturbation simulations, especially when the two ligands to compare require different amounts of water molecules being buried in the active site. In alchemical approaches, the process of binding and unbinding is not simulated, resulting in buried water molecules being unable to leave or enter the active site to correctly fulfil the precise water networks required for each ligand under study, even when using relatively long simulations (50 ns per λ window).^7,8^ One simple solution would be to perturb the required number of water molecules alongside the ligand. However, in order to be able to do that, one must know the required amount of water molecules for each of the ligands before starting the simulations. Having this kind of knowledge about the system a priori might not be possible since there is no simple way, other than chemical intuition, to decide the amount of water molecules required without a crystal structure, and even with a crystal structure, the presence of mobile water molecules may not be detected.^34^

In this work, we have described a new use of the AEDS method to quickly and accurately probe possible hydration sites in a system-independent manner. By using an AEDS water molecule resulting from the combination of a water molecule and a dummy particle, one can infer the likelihood of the water molecule being present at every given location by looking at the amount of time spent in each of the two states, acting in a similar manner to a chemostat. This information can then be used to design and set up further simulations for the system without relying on any additional information. Additionally, the AEDS water can even be used alongside other alchemical methods to have a dynamic water molecule that adapts to the ligand being perturbed, producing the same free energies as one would obtain by perturbing both the ligand and the necessary water molecules. The described AEDS water screening approach can be used in a system-independent manner without requiring to perform additional search runs since the used parameters are only dependent on the water molecule type. In this work, we have used AEDS to study the fractional occupancy of up to two SPC water molecules, but the described methodology could be easily expanded to other water models and to study additional water molecules by either creating a reference AEDS state with more water molecules or randomly selecting the water molecule to probe, which should also lead to the correct ensemble. Probing more than a few water molecules in the active state would become particularly relevant when computing full binding free energies rather than RBFE differences between compounds.

This AEDS water approach works in a different manner than that of the other methods developed to deal with the water sampling issue, which makes direct comparisons difficult.^31^ Instead of using Monte Carlo moves to place water molecules in a predefined area, AEDS allows for constant fluctuation of predefined putative water molecules of interest, with virtually no impact in the performance when compared to a conventional MD of the same system. The use of AEDS waters offers both advantages and disadvantages when compared to those methods. On one hand, AEDS is not able to place new water molecules in the binding site, which means that a previous examination of the system is required to decide how many hydration sites need to be probed. On the other hand, AEDS allows for the comparison of the likelihood of the water molecule to be in a given hydration site in short time scales while also quickly adapting to other molecular motions as seen, for example, in the case of the ligand Cumming-4j.^42^ Additionally, since AEDS uses a boosting potential to accelerate the free-energy landscape of the AEDS molecule with its surroundings, the frequency in which molecular events of interest are observed will also be increased.^37^ Overall, the use of AEDS waters has the potential to aid in the rational design of ligands by allowing to probe putative hydration sites and to be combined with other free-energy techniques.

## Data Availability

All topological information, initial structures, input files, and equilibrated coordinates are made available in the Supporting Information. All simulations were performed with the GROMOS suite of programs for molecular simulations, which is available free of charge at www.gromos.net.

## Supporting Information Available

Acceleration parameters used for the AEDS water with their respective validation, convergence plots for all the water probing simulations, free energies of all paths, molecular topology files, initial structures, input files, and equilibrated coordinate files for all relevant simulations (PDF)
All topological information, initial structures, input files, and equilibrated coordinates for TI–AEDS (ZIP)
