# Supplementary material for: Accelerated Enveloping Distribution Sampling to Probe the Presence of Water Molecules
Source: J Chem Theory Comput. 2023 May 11;19(11):3379–90. doi: 10.1021/acs.jctc.3c00109 (PMC10269333; doi:10.1021/acs.jctc.3c00109)
Supplement: Supplementary file 1 — ct3c00109_si_001.pdf [file ct3c00109_si_001.pdf]

Supplementary Information Accelerated enveloping distribution sampling to probe the presence of water molecules

Oriol Gracia Carmona,[a,†], Michael Gillhofer[a,†], Lisa Tomasiak[a], Anita De Ruiter[a], Chris Oostenbrink[*a,b]*

a. *Institute for Molecular Modeling and Simulation, Department of Material Sciences and Process Engineering, University of Natural Resources and Life Sciences, Vienna. Muthgasse 18, 1190 Vienna, Austria*

b. *Christian Doppler Laboratory for Molecular Informatics in the Biosciences, University of Natural Resources and Life Sciences, Vienna. Muthgasse 18, 1190 Vienna, Austria*

† *Oriol Gracia Carmona and Michael Gillhofer contributed equally.*

* *Corresponding author: chris.oostenbrink@boku.ac.at*

AEDS water parameters

	ONE AEDS WATER	TWO AEDS WATERS	OPTIMIZED LIG A	OPTIMIZED LIG B
**EMAX**	14 kJ/mol	48.4 kJ/mol	70 kJ/mol	70 kJ/mol
**EMIN**	-80 kJ/mol	-165.4 kJ/mol	-100 kJ/mol	-100 kJ/mol
**ΔF_1**	0 kJ/mol	0 kJ/mol	0 kJ/mol	0 kJ/mol
**ΔF_2**	-18.35 kJ/mol	-53.7 kJ/mol	35 kJ/mol	-25 kJ/mol
**ΔF_3**	-	-5.2 kJ/mol	-	-
**ΔF_4**	-	-5.2 kJ/mol	-	-

Table S1. Offsets and acceleration range used for all the AEDS water probing and AEDS – TI simulations. For the cases in which one water molecule is perturbed, ΔF_1 corresponds to the offset used for the coupled state and ΔF_2 for the dummy state. For the case with two water molecules perturbed ΔF_1 corresponds to the offset used for the state with both water molecules coupled, ΔF_2 the double dummy state and ΔF_3, ΔF_4 the states in which only one water molecule is present. The columns 'optimized lig A' (Woodhead-1) and 'optimized lig B' (Woodhead-2,)[1] correspond to the parameters used at both λ windows of the AEDS-TI simulations with optimized offsets. In these, ΔF_1 corresponds to the offset used for the coupled state and ΔF_2 for the dummy state.

AEDS water parameter validation

The obtained parameters for one AEDs water and two AEDS waters, described in table SI 1, were validated by running a 100 ns simulation in solvent. The obtained parameters produced close to equal sampling for each of the end-state combinations with frequent shifts between states, Table SI 2.

	ONE AEDS WATER		TWO AEDS WATERS	
	Sampling	Life times	Sampling	Life times
**NO WATERS**	40.4 %	8.1 ps	19.6 %	2.6 ps
**WATER 1**	59.6 %	5.5 ps	23.1 %	4.0 ps
**WATER 2**	-	-	23.5 %	4.0 ps
**2 WATERS**	-	-	33.7 %	11.5 ps

Table S2. Sampling times and average life times of each of the end states belonging to the reference AEDS state with one water molecule and two water molecules. The final sampling times for each water molecule are close to 50%, with 59.6 % for the single AEDS water and 56.8 %, 57.2 % for the reference state with two AEDS waters respectively.

Water probing convergence

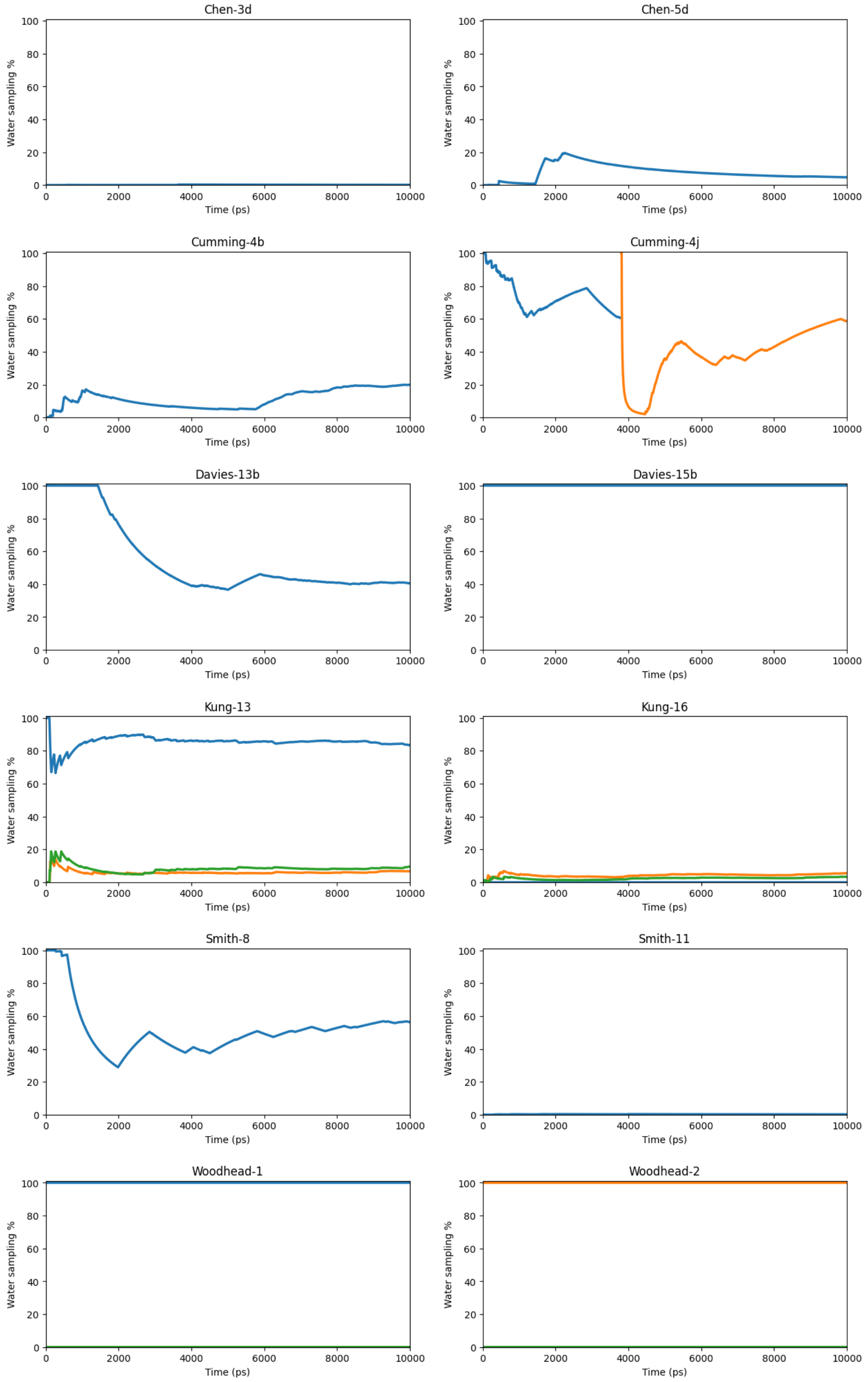

Figure S1. Forward cumulative average plots for the water sampling of each of the ligands under study[1–6] with their respective proteins over 10-ns simulations. For ligand Cumming-4j,[2] the two different conformations of the ligand are shown by separate time traces, with the conformation observed in the crystal structure in blue and the conformation with the pyridine ring flipped in orange. For ligands Kung-13, Kung-16[5], Woodhead-1, Woodhead-2,[1] since there are multiple water molecules, the different combinations are shown by separate time traces, the blue line corresponds to both water molecules being present, the orange line correspond to the first water molecule being present and the green line correspond to the second water molecule being present.

Free energies

	EXTENDED TI	TI	TI - AEDS	TI – AEDS OPTIMIZED
$\Delta G_{A \rightarrow B\ solvent}$	16.5 ± 1 ***	26.3± 0	26.3± 0	26.3 ± 0
$\Delta G_{AW \rightarrow BW\ prot}$	35.7 ± 1.2 ***	43.2 ± 1.2	43.4 ± 1.1*	45.3 ± 1.6 *
**$\Delta G_{AW \rightarrow BD\ prot}$**	**57.8 ± 0.6 *** **	**59.9 ± 1.6**	**60.2 ± 1.4**	**58.9 ± 1.5**
$\Delta G_{AD \rightarrow BD\ prot}$	-	20.8 ± 1.3	22.6 ± 1.1 *	18.5 ± 1.4 *
$\Delta G_{ADD \rightarrow BDD\ prot}$	10.5 ± 0.1	-	-	-
$\Delta G_{AW \rightarrow BDD\ prot}$	86 ± 0.3	-	-	-
$\Delta G_{AR \rightarrow BR\ prot}$	-	-	31.6 ± 1.4 **	40.8 ± 1.4 **
$\Delta G_{AW \rightarrow AR\ prot}$	-	-	0 ± 0 **	4.8 ± 0.5 **
$\Delta G_{AR \rightarrow AD\ prot}$	-	-	65.4 ± 2.2 **	38.2 ± 1.2 **
$\Delta G_{BW \rightarrow BR\ prot}$	-	-	-41.7 ± 2.3 **	3.1 ± 0.9 **
$\Delta G_{BR \rightarrow BD\ prot}$	-	-	28.6 ± 0 **	13.3 ± 0.7 **
$\Delta\Delta G_{AW \rightarrow BW}$	19.2 ± 1.5	16.9 ± 1.2	17.1 ± 1.1	19 ± 1.6
$\Delta\Delta G_{AW \rightarrow BD}$	**6.9 ± 1.1**	**6.8 ± 1.6**	**7.1 ± 1.4**	**5.8 ± 1.5**
$\Delta\Delta G_{AD \rightarrow BD}$	-	-5.5 ± 1.3	-3.7 ± 1.1	-7.8 ± 1.4
$\Delta\Delta G_{ADD \rightarrow BDD}$	-11 ± 1	-	-	-
$\Delta\Delta G_{AW \rightarrow BDD}$	-1.2 ± 1.2	-	-	-

* Predicted values trough reweighting

** Values are not directly comparable since the reference AEDS state used is different

*** Perturbation topology is different from the other simulations

Table S3. Free energies obtained for perturbing Woodhead-1 to Woodhead-2 in the HSP90 protein and in solvent for the extended TI simulations, TI simulations suing the same setting as the AEDS-TI ones, AEDS-TI simulation with the universal water parameters and TI – AEDS with optimized acceleration parameters. Lig A correspond to Woodhead-1 and lig B correspond to Woodhead-2.[1] The W indicates that both crystallographic water molecules are present, D indicates that water 2144 is in dummy state and DD indicates that both crystallographic water molecules are in dummy state. The final ΔΔG contain the corrections to account for any distance restraints as described in the manuscript. Values in bold correspond to the perturbation using the number of water molecules that AEDS found to be relevant. The experimental free energy is 8.4 kJ/mol.[1] All units are in kJ/mol.

References

(1) Woodhead, A. J.; Angove, H.; Carr, M. G.; Chessari, G.; Congreve, M.; Coyle, J. E.; Cosme, J.; Graham, B.; Day, P. J.; Downham, R.; Fazal, L.; Feltell, R.; Figueroa, E.; Frederickson, M.; Lewis, J.; McMenamin, R.; Murray, C. W.; O'Brien, M. A.; Parra, L.; Patel, S.; Phillips, T.; Rees, D. C.; Rich, S.; Smith, D.-M.; Trewartha, G.; Vinkovic, M.; Williams, B.; Woolford, A. J.-A. Discovery of (2,4-Dihydroxy-5-Isopropylphenyl)-[5-(4-Methylpiperazin-1-Ylmethyl)-1,3-Dihydroisoindol-2-Yl]Methanone (AT13387), a Novel Inhibitor of the Molecular Chaperone Hsp90 by Fragment Based Drug Design. *J Med Chem* **2010**, *53* (16), 5956–5969. https://doi.org/10.1021/jm100060b.

(2) Cumming, J. N.; Smith, E. M.; Wang, L.; Misiaszek, J.; Durkin, J.; Pan, J.; Iserloh, U.; Wu, Y.; Zhu, Z.; Strickland, C.; Voigt, J.; Chen, X.; Kennedy, M. E.; Kuvelkar, R.; Hyde, L. A.; Cox, K.; Favreau, L.; Czarniecki, M. F.; Greenlee, W. J.; McKittrick, B. A.; Parker, E. M.; Stamford, A. W. Structure Based Design of Iminohydantoin BACE1 Inhibitors: Identification of an Orally Available, Centrally Active BACE1 Inhibitor. *Bioorg Med Chem Lett* **2012**, *22* (7), 2444–2449. https://doi.org/10.1016/j.bmcl.2012.02.013.

(3) Chen, J. M.; Xu, S. L.; Wawrzak, Z.; Basarab, G. S.; Jordan, D. B. Structure-Based Design of Potent Inhibitors of Scytalone Dehydratase: Displacement of a Water Molecule from the Active Site. *Biochemistry* **1998**, *37* (51), 17735–17744. https://doi.org/10.1021/bi981848r.

(4) Smith, C. R.; Dougan, D. R.; Komandla, M.; Kanouni, T.; Knight, B.; Lawson, J. D.; Sabat, M.; Taylor, E. R.; Vu, P.; Wyrick, C. Fragment-Based Discovery of a Small Molecule Inhibitor of Bruton's Tyrosine Kinase. *J Med Chem* **2015**, *58* (14), 5437–5444. https://doi.org/10.1021/acs.jmedchem.5b00734.

(5) Kung, P.-P.; Sinnema, P.-J.; Richardson, P.; Hickey, M. J.; Gajiwala, K. S.; Wang, F.; Huang, B.; McClellan, G.; Wang, J.; Maegley, K.; Bergqvist, S.; Mehta, P. P.; Kania, R. Design Strategies to Target Crystallographic Waters Applied to the Hsp90 Molecular Chaperone. *Bioorg Med Chem Lett* **2011**, *21* (12), 3557–3562. https://doi.org/10.1016/j.bmcl.2011.04.130.

(6) Davies, N. G. M.; Browne, H.; Davis, B.; Drysdale, M. J.; Foloppe, N.; Geoffrey, S.; Gibbons, B.; Hart, T.; Hubbard, R.; Jensen, M. R.; Mansell, H.; Massey, A.; Matassova, N.; Moore, J. D.; Murray, J.; Pratt, R.; Ray, S.; Robertson, A.; Roughley, S. D.; Schoepfer, J.; Scriven, K.; Simmonite, H.; Stokes, S.; Surgenor, A.; Webb, P.; Wood, M.; Wright, L.; Brough, P. Targeting Conserved Water Molecules: Design of 4-Aryl-5-Cyanopyrrolo[2,3-d]Pyrimidine Hsp90 Inhibitors Using Fragment-Based Screening and Structure-Based Optimization. *Bioorg Med Chem* **2012**, *20* (22), 6770–6789. https://doi.org/10.1016/j.bmc.2012.08.050.
